# Compositional and Functional Adaptations of Intestinal Microbiota and Related Metabolites in CKD Patients Receiving Dietary Protein Restriction

**DOI:** 10.3390/nu12092799

**Published:** 2020-09-12

**Authors:** I-Wen Wu, Chin-Chan Lee, Heng-Jung Hsu, Chiao-Yin Sun, Yuen-Chan Chen, Kai-Jie Yang, Chi-Wei Yang, Wen-Hun Chung, Hsin-Chih Lai, Lun-Ching Chang, Shih-Chi Su

**Affiliations:** 1 Department of Nephrology, Chang Gung Memorial Hospital, Keelung 20401, Taiwan; fliawu@yahoo.com (I.-W.W.); leefang@cgmh.org.tw (C.-C.L.); r5267@cgmh.org.tw (H.-J.H.); fish3970@gmail.com (C.-Y.S.); eva90156@cgmh.org.tw (K.-J.Y.); 2 College of Medicine, Chang Gung University, Taoyuan 33305, Taiwan; cyc2356@cgmh.org.tw (Y.-C.C.); cwyang00@gmail.com (C.-W.Y.); 3 Kidney Research Center, Department of Nephrology, Chang Gung Memorial Hospital, Linkuo 33305, Taiwan; 4 Whole-Genome Research Core Laboratory of Human Diseases, Chang Gung Memorial Hospital, Keelung 20401, Taiwan; chung1@cgmh.org.tw; 5 Department of Medical Biotechnology and Laboratory Science and Microbiota Research Center, College of Medicine, Chang Gung University, Taoyuan 333, Taiwan; hclai@mail.cgu.edu.tw; 6 Department of Mathematical Sciences, Florida Atlantic University, Boca Raton, FL 33431, USA; 7 Department of Dermatology, Drug Hypersensitivity Clinical and Research Center, Chang Gung Memorial Hospital, Linkou 33305, Taiwan

**Keywords:** bile acids, chronic kidney disease, gut microbiome, low protein diet, short-chain fatty acids, uremic solute

## Abstract

The relationship between change of gut microbiota and host serum metabolomics associated with low protein diet (LPD) has been unraveled incompletely in CKD patients. Fecal 16S rRNA gene sequencing and serum metabolomics profiling were performed. We reported significant changes in the β-diversity of gut microbiota in CKD patients having LPD (CKD-LPD, *n* = 16). We identified 19 genera and 12 species with significant differences in their relative abundance among CKD-LPD patients compared to patients receiving normal protein diet (CKD-NPD, *n* = 27) or non-CKD controls (*n* = 34), respectively. CKD-LPD had a significant decrease in the abundance of many butyrate-producing bacteria (family *Lachnospiraceae* and *Bacteroidaceae*) associated with enrichment of functional module of butanoate metabolism, leading to concomitant reduction in serum levels of SCFA (acetic, heptanoic and nonanoic acid). A secondary bile acid, glyco λ-muricholic acid, was significantly increased in CKD-LPD patients. Serum levels of indoxyl sulfate and p-cresyl sulfate did not differ among groups. The relationship between abundances of microbes and metabolites remained significant in subset of resampling subjects of comparable characteristics. Enrichment of bacterial gene markers related to D-alanine, ketone bodies and glutathione metabolism was noted in CKD-LPD patients. Our analyses reveal signatures and functions of gut microbiota to adapt dietary protein restriction in renal patients.

## 1. Introduction

Dietary protein restriction is commonly recommended in moderate to advanced chronic kidney disease (CKD) patients; however, the effectiveness of consumption of low protein diet (LPD, <0.8 g/kg body weight/day) to preserve renal function remains an area of continuous debate [1,2,3]. Low protein intake can ameliorate proteinuria, through the regulation of intraglomerular pressure and angiotensin pathway, decrease sodium loading and reduce urea and nitrogenous wastes; consequently, limits uremia [4,5]. However, nutritional imbalance and protein energy wasting represent key concerns, in regards to dietary adherence and surveillance, particularly in special populations (such as children in growth age or elderly patients). While the implementation of very low protein diet (VLPD, 0.4–0.6 g/kg body weight/day) supplemented with ketoanalogues amino acids can retard renal progression without development of caloric or nutritional detriments [1,2]; however, experimental model has demonstrated that VLPD enhance inflammation, malnutrition and aortic calcification [6]. The precise benefit and pathophysiology of LPD on gut-renal axis remain partially elucidated in CKD patients.

The diet constitutes the substrate for intestinal fermentation affecting the gut microbiota and leading to production of diverse metabolites causing metabolic disarrangements [7,8]. Little is known about the nutrient-associated microbiome changes in CKD patients, considering all the intestinal dysbiosis and dietary restrictions presented in renal patients. Significant reduction in serum levels of p-cresyl sulfate (pCS) and changes of gut microbiota were found in moderate CKD patients receiving 6-month of LPD. However, no clustering of pattern was observed in the gut microbiota of patients consuming LPD or free diet [9]. The implementation of VLPD was associated with a reduced abundance of Proteobacteria and increased level of Blautia, Faecalibacterium and Coprococcus and Roseburia species and the concomitant reduction in serum levels of both indoxyl sulfate (IS) and pCS, compared to those patients receiving free diet [10]. Despite significant changes in the abundance of selected intestinal microbes associated with dietary protein deprivation, however, modifications of bacterial functional capability and other related metabolites secondary to this adaptive change of gut microbiota remain unclear. Hence, in the present study, we aim to explore the change in intestinal microbiota, related metabolomic profiling and bacterial functional capability associated with LPD in CKD patients.

## 2. Materials and Methods

### 2.1. Subjects and Settings

Forty-three CKD patients and 34 matched normal non-CKD controls (in terms of age, gender, and presence of diabetes or hypertension) were recruited from Chang Gung Memorial Hospital, Keelung, Taiwan (Figure S1). CKD was diagnosed if having either proteinuria or an estimated glomerular filtration rate (eGFR, calculated by applying simplified Modification of Diet in Renal Disease equation) of less than 60 mL/min/1.73 m2 in two separate occasions. From the CKD patients, 16 patients received LPD (<0.8 g/kg body weight/day) and 27 patients received a normal protein diet (NPD, 1 g/kg body weight/day) for 3 months. Patients were excluded from study if receiving dialysis therapy, or having renal transplant, cardiovascular disease, active infection, malignancy, liver cirrhosis, intestinal operation, irritable bowel syndrome, pregnancy, or concomitant use of probiotics, prebiotics or antibiotics. Patients receiving vegan or vegetarian diets were excluded to avoid distortion on the dietary pattern of the entire cohort. The participants were not permitted to take any supplement containing probiotics, such as yogurt, within 7 days before sample collection. All fasting plasma and fresh stools were appropriately collected at the end of the third month and stored at −80 °C until analysis. A minimal of 65 total samples was found to have a study power of 0.95 and ⍺-error probability of 0.05 in a 3-group design (non-CKD control, NPD and LPD groups), based on effect size of 50% and significance level at 0.05 under two-tail analysis. A study number of 77 patients were justified by sample size calculation statement. This study was conducted in adherence to the Declaration of Helsinki and approved by the Institutional Review Board at Chang Gung Memorial Hospital (IRB: 104-0973C, 104-5478B, 102-5507A3, 201802061B0, 201802245B0 and 201900167B). The informed consents were obtained from all patients (Clinical Trials gov.NCT04300387).

### 2.2. Low Protein Diet and Compliance

A single dietitian instructed the LPD intake for all patients. All patients were standardized to receive a 3-meal dietary pattern, and both animal or vegetal sources of protein could be chosen. Daily protein intake of <0.8 g/kg body weight/day, with 80% of high biological value protein, such as meat, poultry, fish, eggs, milk, cheese or soy bean, was recommended for 3 months. Food records and 24-h recall were used to assess dietary intake. In addition, 24-h urine was collected for every patient at the end of the third month to assess their compliance to LPD, and the estimated protein intake (g/day) was calculated by the following formula: 6.25 × [Urine urea nitrogen (g/day) + 30 mg/kg/day × Weight (kg)]. The same dietitian reviewed the diet of all non-CKD and CKD-NPD patients to ensure adequate protein and caloric intake.

### 2.3. Targeted Metabolomics Profiling of Gut-Producing Metabolites

Concise methodology of target metabolomic profiling was described in our previous report [11,12]. Briefly, 250 μL of internal standard solution containing 10% H2SO4 (Sigma) and 20 mg/L 2-methylvaleric acid (Dr. Ehrenstorfer GmbH, Augsburg, Germany) was added to 150 μL of serum samples, for profiling of 11 short-chain fatty acids (SCFA) and medium-chain fatty acids (MCFA, Table S1) by GC-MS analysis using an Agilent 7890B gas chromatograph system coupled with an Agilent 5977B mass spectrometer. For the analysis of 41 circulating bile acids (Table S2), 100 μL of serum sample was mixed with 400 μL of extract solvent (acetonitrile-methanol, 1:1, containing 0.1% formic acid) to extract the supernatant for subsequent UHPLC-MS/MS analysis. UHPLC separation was performed in an Agilent 1290 Infinity series UHPLC System, equipped with a Waters ACQUITY UPLC BEH C18 column (150 × 2.1 mm, 1.7 μm, Waters, Agilent Technologies, Santa Clara, CA, USA). The MS analysis was conducted by using a Q Exactive Focus mass spectrometer (Thermo Fisher Scientific, Waltham, MA, USA). Circulating pCS and IS (free and protein-bound fractions) were analyzed with UPLC-MS/MS (Milford, MA, USA). Concentrations of free pCS and IS were measured in serum ultrafiltrates by using AmicoUltra 30 K filter (Millipore, Burlington, MA, USA). Samples were deproteinized by addition of acetonitrile. Chromatographic separation was performed at 30 °C using Acquity UPLC BEHC 18 column (2.1 × 100 mm). The analytes were quantified with Waters Acquity UPLC Xevo TQ-S operating in negative electrospray ionization and multiple reaction monitoring mode [12,13].

### 2.4. Fecal 16S rRNA Gene Sequencing and Functional Prediction of Bacterial Gene

The FastDNA SPIN Kit for Feces (MP Biomedical, LLC) was used to extract fecal bacterial DNA. The 16S rRNA gene sequencing, data processing and analysis followed the same pipeline of our previous work [13]. In brief, we applied polymerase chain reaction (PCR) to amplify the variable region 4 (V4) of the gene that encodes for 16S rRNA in bacteria for further sequencing on an Illumina HiSeq 2500 platform. The processed sequencing reads (effective tags) were clustered into operational taxonomic units (OTU) at 97% sequence identity using UPARSE [14], and taxonomy classification was assigned according to the information retrieved from the SILVA database [15]. Chao1 index was used to determine the species richness or α-diversity. For evaluating β-diversity, Bray–Curtis dissimilarities were estimated to evaluating β-diversity [16]. Non-metric dimensional scaling (NMDS) was conducted using the weighted correlation network analysis in R software. The phylogenetic reconstruction of unobserved states (PICRUSt) software was conducted to dissect the functional composition of metagenomes predicted from 16S rRNA data [17]. Finally, we precomputed for gene content prediction using table of gene copy numbers for each gene family in each sequenced bacterial and archaeal genome based on the IMG database [18] and phylogenetic tree from the Greengenes database [19].

### 2.5. Statistics Analysis

Descriptive statistics were expressed as the mean, median or frequency. Kolmogorov–Smirnov method was used to test the normality of numerical variables. Student’s *t*-test, nonparametric median test or Kruskal–Wallis test were applied to measure differences in clinical indices among groups. Chao1 index was analyzed using Kruskal–Wallis test and Bray–Curtis distance between groups was calculated by Wilcoxon rank sum test. The discrimination in community composition between groups was determined by analysis of similarities (ANOSIM) of UniFrac parameters using 999 permutations in each test. Spearman’s correlation was used to determine the association of major genera (>0.1% abundance and present in >90% of samples) with daily protein intake. Significant differences in the relative abundance of the taxa among three groups were compared at the genus and species level using Kruskal–Wallis test and the post-hoc comparison between two groups by Dunn’s test [20]. The linear discriminant analysis (LDA) of effect size (LEfSe) analysis was performed to evaluate statistically significant taxa. The non-parametric factorial Kruskal–Wallis test, Wilcoxon rank sum test and LDA were employed to identify differentially abundant taxa between two metadata classes. Random Forests were used to identify important taxa for classifying CKD-LPD [21], which ranked OTUs based on their ability to discriminate among the groups, while taking into account the complex interrelationships in high dimensional data. Student’s t test was applied to denote differences in relative abundance of predicted microbial genes related to metabolism between groups. Data were analyzed using SPSS 22.0 for Windows XP (SPSS Inc., Chicago, IL, USA). All reported p values were two-tailed, and a *p* value of <0.05 was considered significant.

## 3. Results

### 3.1. Subject Characteristics

The Table 1 lists the baseline characteristics of participants. The patients receiving LPD (*n* = 16) were more likely to have lower eGFR, hemoglobin and serum albumin than the NPD patients (*n* = 27, Table 1). The estimated protein intake was significantly lower in the LPD than NPD patients or the normal controls, indicating good compliance to LPD instruction. No use of phosphate binders or carbonaceous oral adsorbents (AST-120) were recorded in overall patients. Scarce number of patients received potassium chelator (1 and 2 patients in CKD-NPD and CKD-LPD group, respectively).

### 3.2. Changes of Microbial Composition and Diversity in CKD Patients Receiving LPD

**Figure 1:**
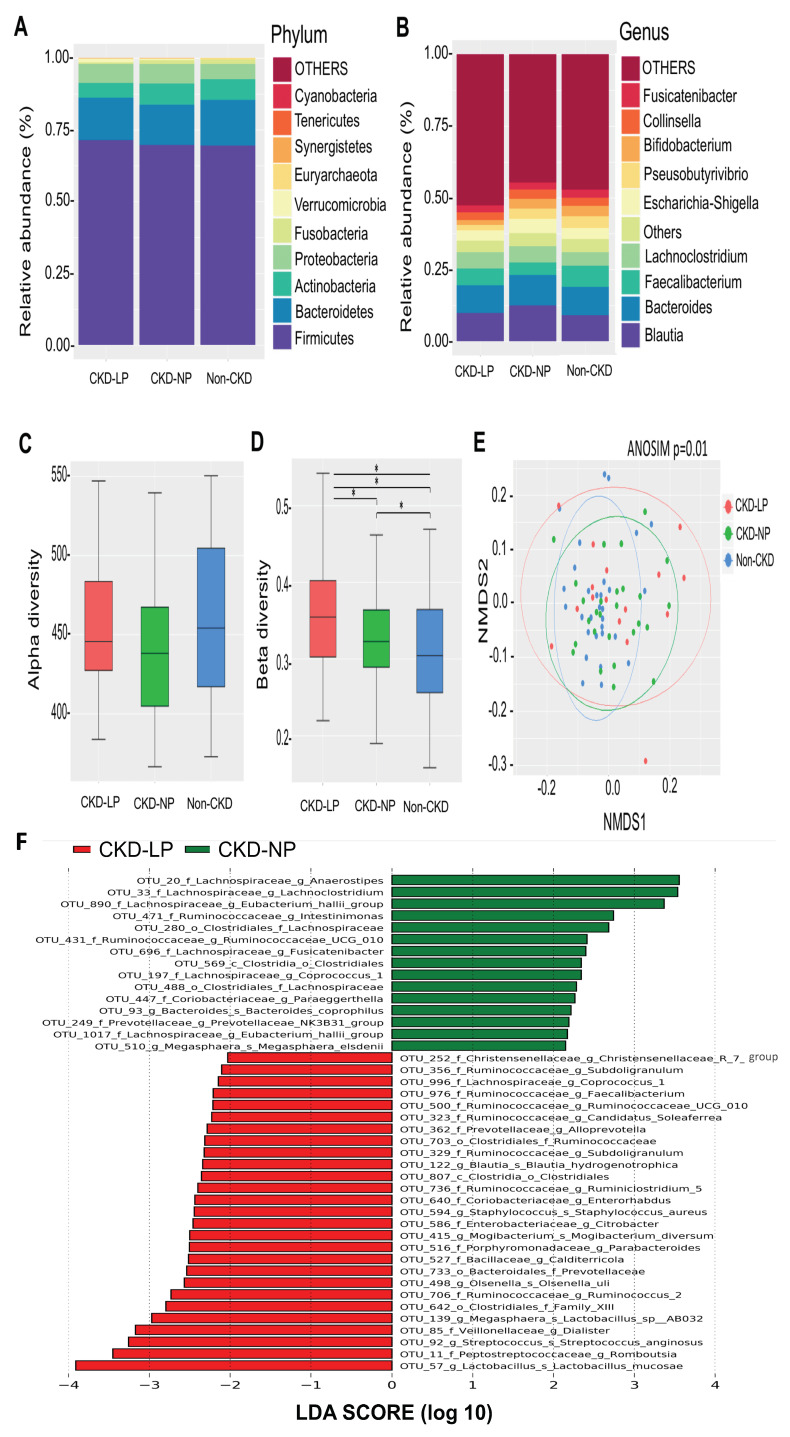
Comparisons of gut microbiota composition and diversity in non-CKD controls and CKD patients receiving LPD or NPD. (**A**) The distribution of top 10 phyla and top 10 genera (**B**) detected among groups. (**C**) α- diversity (Chao 1) and (**D**) β-diversity (Bray–Curtis similarity index) of gut microbial communities among groups. The box-plot shows the median, the 25th, and the 75th percentile in each group. *, *p* < 0.001 (**E**) Nonmetric multidimensional scaling (NMDS) ordination based on weighted UniFrac parameters of intestinal microbial communities among groups. Significant sample-to-sample dissimilarities refer to analysis of similarity (ANOSIM, *p* = 0.01) test for discrimination in community composition among groups. (**F**) Bacterial taxa that best characterize each group were determine by applying linear discriminant analysis of effect size (LEfSe) on OTU tables. LP, low protein diet; NP, normal-protein diet.

The Taxonomic analysis (at phylum level) revealed an increase in Firmicute and Bacteroidetes and a decrease in Actinobacteria in CKD-LPD patients compared to those patients receiving NPD (Figure 1A). Significant compositional change was also noted at lower taxonomic level among the three groups (Figure 1B). There were no differences in α-diversity among the three groups (Figure 1C). Moreover, analyses of sample-to-sample dissimilarities in bacterial community structures (β-diversity, Figure 1D) demonstrated that the gut microbiome of CKD patients receiving LPD or NPD clustered separately from that of the non-CKD controls (Figure 1E, ANOSIM, *p* = 0.01), indicating variations in gut microbiome throughout different dietary regimens among CKD patients.

Since disturbance in the relative abundance of gut microbiome was observed, we explored the change of specific gut microorganism at the genus and species level associated with LPD vs. NPD. Using rigorous criteria (>0.1% abundance and present in >90% of samples with threshold for selection to 2.5 or 3.0 at LDA score), 19 genera and 12 species with significant differences in the relative abundance among three groups were identified (Table 2).

Moreover, extensive analyses were conducted to disclose microbial taxa associated with LPD in CKD patients. We predicted the biomarkers for LPD vs. NPD group by taking statistical significance and biological consistency into consideration using LEfSe (Figure 1F). Among these microorganisms, a significant relationship was also observed between daily protein intake and the relative abundance of intestinal microbiome. The relative abundances of Anaerostipes and Eubacterium hallii group were positively correlated, and those abundances of Calditerricola, Streptococcus anginosus, Lactobacillus mucosa and Clostridium paraputrificum were negatively correlated with protein intake in the overall patients as well as the only CKD cohort (Table 3).

Furthermore, we applied the Random Forests analysis to classify the best discriminatory taxa associated with LPD, using the overall (1024 OTU, Figure S2A), only the genus (246 OTU, Figure S2B) or the species level (180 OTU, Figure S2C) microbiota profiles. Consistently, many OTUs were identified to be capable of categorizing CKD-LPD from CKD-NPD or the controls, based on multiple sensitivity analyses.

### 3.3. Changes of Targeted Metabolomics Profiling in CKD Patients Receiving LPD

**Figure 2:**
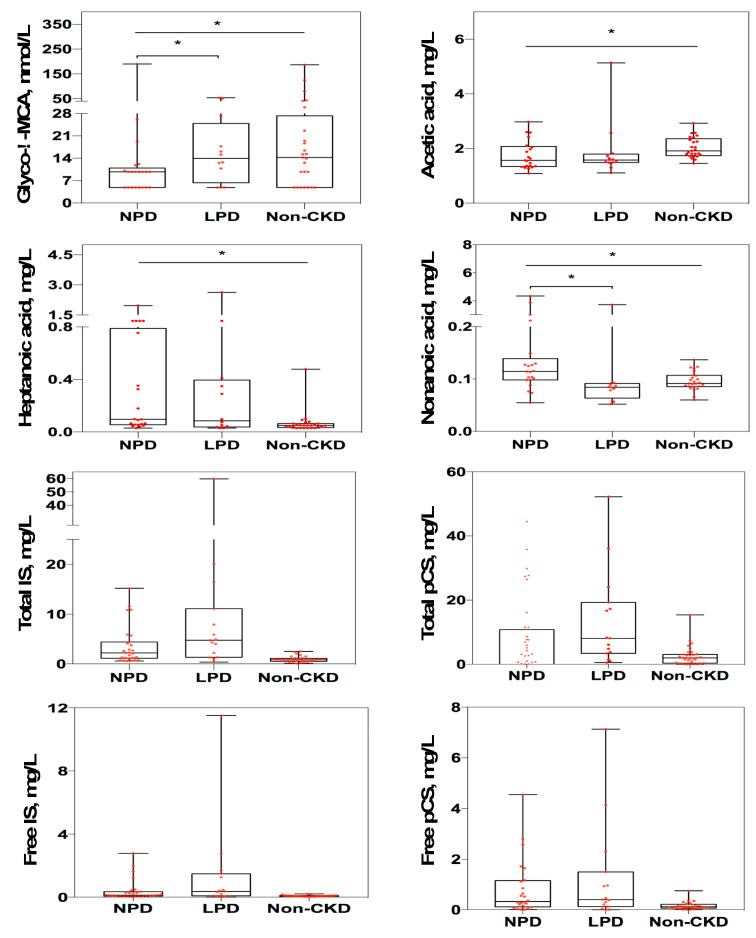
Changes in circulating metabolite concentration associated with LPD in CKD patients. Levels of metabolites among different groups were analyzed by Wilcoxon rank sum test. The box-plot shows the median, the 25th, and the 75th percentile in each group. *, *p* < 0.05. LPD, low protein diet; NPD, normal-protein diet; CKD, chronic kidney disease; glyco-λ-MCA, glyco-λ-muricholic acid; IS, indoxyl sulfate; pCS, p cresyl-sulfate.

To examine CKD-LPD associated changes in the host-microbe-derived metabolites, we conducted targeted metabolomic profiling of 11 saturated fatty acids (Table S1), 41 bile acids (Table S2), and two uremic solutes (IS and pCS). The concentrations of bile acid, glyco λ-muricholic acid, and three fatty acids (acetic acid, heptanoic acid and nonanoic acid) were significantly different among three groups (Figure 2). Compared to CKD-NPD patients, the serum levels of glyco λ-muricholic acid were significantly increased (*p* = 0.027) but the levels of nonanoic acid were decreased (*p* = 0.002) in CKD-LPD patients. The serum concentration of both IS (total form, *p* = 0.054; free form, *p* = 0.105) and pCS (total form, *p* = 0.548; free form, *p* = 0.462) did not differ between CKD-LPD vs. CKD-NPD, respectively (Figure 2).

To control as possible the confounding effect of baseline difference of renal function of patients on the outcome of study, we have re-sampled a subset of patients from stratified sampling by CKD stage with individualized match to age. Stratified sampling by CKD stage identified 30 patients. Only 28 CKD patients were included in the subset analysis after individualized matched pare of ±1 y/o of age. Significant differences on the relative abundances of specific microbes and two metabolites (glyco-λ-muricholic acid and Nonanoic acid) were presented between CKD-LPD vs CKD-NPD patients of the resampling subset (Table 4).

### 3.4. Functional Prediction of Change of Intestinal Microbiota Associated with LPD in CKD Patients

**Figure 3:**
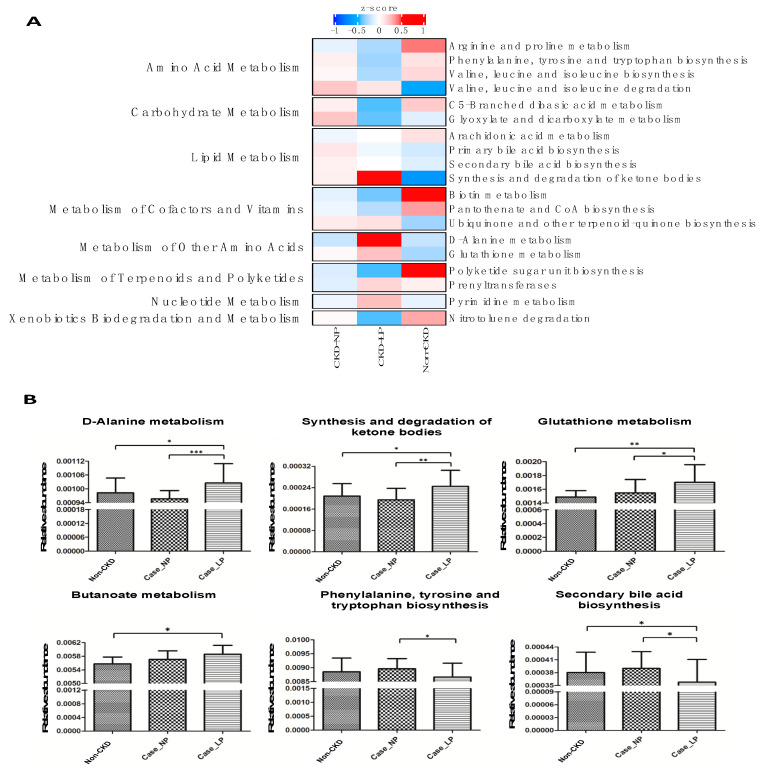
Prediction of microbial gene functions among groups. (**A**) Pathway enrichment for KEGG metabolism was inferred by PICRUSt. Differences in relative abundance of predicted microbial genes related to the metabolism among groups. (**B**) Changes of specific pathway modules associated with LP in CKD patients. Differences in relative abundances of predicted microbial genes among LP vs. NP were analyzed using Student’s t test. *, *p* < 0.05; **, *p* < 0.01; ***, *p* < 0.001. LP, low protein diet; NP, normal-protein diet; CKD, chronic kidney disease.

To gain an insight into the functionality of fecal microbiota associated with LPD, we inferred the functional profile of bacterial communities by PICRUSt [17]. With a focus on pathways relevant to microbial metabolism, we found that in addition to the difference detected in bacterial composition and diversity, several pathway modules associated with metabolism of amino acid, carbohydrate and lipid were differentially enriched between CKD-LPD vs. CKD-NPD (Figure 3A). Consistently, the changes of genetic markers assigned to metabolism of butanoate (prototype of SCFA) and biosynthesis of secondary bile acids corresponded to the variation of the serum levels of aforementioned metabolites observed in CKD-LPD patients (Figure 3B). Although we did not find differences of the serum levels of IS and pCS between CKD-LPD vs. NPD patients, notable reductions on the microbial pathway related to the biosynthesis of phenylalanine, tyrosine and tryptophan were observed in former group of patients. Furthermore, microbial genes related to the metabolism of D-alanine, synthesis and degradation of ketone bodies, and metabolism of glutathione were differentially enriched between the two dietary regimens of CKD patients (Figure 3B). Collectively, data shown in the present study indicated compositional and functional variations of gut microbiota associated with LPD in CKD patients. These variations were associated with the serum levels of gut-producing metabolites, such as fatty acids and bile acids. These connections highlight a potential host–microbe–metabolite interaction secondary to dietary protein restriction in CKD patients.

## 4. Discussion

Knowledge on diet–microbiome–metabolite interaction of CKD patients remains mandatory to support long-term dietary interventions, which allows modulation of an individual’s enterotype to preserve renal function. Comprehensive discernments of relationships between change of gut microbiota and serum metabolomic profiling associated with different dietary instruction remain incompletely understood in CKD patients. Here, we reported significant change of composition and diversity of gut microbiota and its associated functional shift in strong association with circulating metabolites in CKD patients receiving LPD. CKD-LPD patients had significant reduction in the relative abundance of many butyrate-producing bacteria (family Lachnospiraceae and Bacteroidaceae) associated with enrichment of functional module of metabolism of carbohydrate, specifically, the butanoate metabolism. The abundances of these microbes were highly correlated with daily protein intake. Consequently, CKD-LPD patients had lower serum levels of acetic acid, heptanoic acid and nonanoic acid. In addition, the serum levels of glyco λ-muricholic acid was significantly increased in patients under dietary protein restriction compared to CKD-NPD. Correspondently, the abundance of microorganism responsible for fermentation of secondary bile acid (family Veillonellaceae, genera Megasphaera) was increased with differential enrichment of microbial gene associated with secondary bile acid biosynthesis. Overall, our analyses reveal signatures and functions of gut microbiota related to dietary protein restriction in CKD patients.

Consistent with our [11] and many other reports [22,23], in which a descending trend in α-diversity was associated with the CKD severity, the decrease in gut microbial diversity between CKD and normal control subjects was also noted in the present study. However, the difference of ⍺-diversity between CKD-LPD and CKD-NPD patients was not as prominent in our investigation as in others [10,24]. In contrast with these reports, our study has demonstrated a strong bacterial community dissimilarity (β-diversity) between patients receiving LPD vs. NPD. The discrepancy observed between studies may in part be explained in the limited sample size between studies and also in the supplementation given to patients having LPD. The addition of inulin [24] and ketoanalogues amino acids [10] may have an influence on the gut microbiota by providing extra substrates for microbial nutrient metabolism compared to our mere LPD intervention.

The SCFAs are gut-derived metabolites produced from fermentation of dietary fiber by anaerobic microbes. Acetate, propionate, and butyrate are the three most common SCFAs and exert many renoprotective properties, such as anti-inflammation, anti-atherosclerosis, anti-oxidative functions [25,26]. Our and other previous investigations have indicated a reduction in levels of SCFA, especially the butyrate, associated with decreased butyrate-producing bacteria (family Lactobacillaceae and Prevotellaceae) in CKD patients [11,27,28]. With the restriction of dietary protein intake, the abundance of many of these bacteria (Pseudobutyrivibrio, Lachnospira, Eubacterium_hallii_group, Roseburia, Coprococcus, Fusicatenibacter, Anaerostipes, Lachnoclostridium and Prevotellaceae_NK3B31) and serum levels of specific SCFA/MCFA (acetic acid, heptanoic acid and nonanoic acid) were lower compared to CKD-NPD or non-CKD controls. In contrast to the decrease in abundance of most of the butyrate-producing bacteria observed in our study, the abundance of Faecalibacterium prausnitzii (a main butyrate-producer) was increased in our and other patients consuming LPD [10] than in CKD-NPD patients; in spite of this increase, however, the abundance of this microbe in CKD-LPD patients remained low compared to the abundance of non-CKD control. The negligible effect of LPD in increasing levels of SCFA in CKD patients may in part be attributed by the severe gut dysbiosis caused by uremic milieu and also by the reduced fiber intake in CKD patients. The relationships between levels of SCFA/MCFA and the outcome of patients receiving dietary protein restriction deserve further study.

The serum concentrations of total bile acids are increased in renal patients because of the reduction in glomerular filtration and derangement of bile acid metabolism secondary to the gut dysbiosis of CKD [29,30]. Increased serum levels of taurocholic acid, taurochenodeoxycholic acid, taurohyocholic acid and tauro α-muricholic acid were associated with death in ESRD patients [30]. Secondary bile acids are derived by gut microbes via the biotransformation of primary bile acids produced in liver. Reciprocally, the bile acid receptor, also known as farnesoid X receptor (FXR), expressed at high concentration in both ileum and liver, can exert negative feedback on the liver production of primary bile acids, in the situation of elevated levels of secondary bile acids [31]. The biological functions of secondary bile acids are pleotropic and remain elusive. They are proposed to have roles on host energy production, intestinal immunity, oxidative damage, colonic carcinogenesis and dysmetabolism, such as diabetes or obesity [31,32,33]. In a germ-free mice model, tauro-conjugated muricholic acids act on FXR of ileum to result on the suppression of bile acid synthesis in the liver [34]. The expansion of glyco λ-muricholic acid observed in CKD-LPD patients may affect the regulation of bile acids burden in adapting change of dysbiosis or of specific dietary pattern in CKD patients; however, the exact roles need further investigation.

Nutritional or iatrogenic therapy alters intestinal microbiota resulting in alleviation of serum levels of IS and pCS in patients receiving LPD, oral vancomycin, prebiotics or probiotics [10,35,36]. The change of tryptophanase-producing bacteria was remarkable in our CKD-LPD patients as well as several studies (family: clostridiacea, ruminococcaceae, lachnospiraceae and genera: roseburia, faecalibacterium) [10,13,28]. Unexpectedly, the serum levels of IS and pCS did not vary in our patients receiving LPD, in spite of significant change of microbiota and gene marker associated with phenylalanine, tyrosine and tryptophan biosynthesis. The difference on the source of dietary protein (red meat or soy bean) can shape gut microbial composition. A Western diet characterized by animal protein and fat was associated with predominance of Bacteroides enterotypes versus the Prevotella enterotype observed in carbohydrates-based diet [8]. The small sample size and the low residual renal function of the CKD-LPD group may also contribute to this divergence. Recently, simultaneous measurements of levels of p-cresol, indol and indol-3-acetic acid in feces, plasma, and urine of different stages of CKD patients found that intestinal generation of these toxins did not contribute to the difference of concentration detected in their serum. The renal tubular clearance represented the key determinant of serum concentration of these solutes [37]. However, the differences on the abundance of selected microbes and other gut-producing metabolites between patients with distinct protein intake remained significant in the resampling subset of subjects having comparable renal function (Table 4). It is likely that LPD might eventually lead to greater uremic symptoms even if rate of GFR progression is slower given the uremic toxin differences. The possibility of renoprotection associated with lowering of gut-producing uremic toxins, induced by the manipulation of dietary protein, remains to be proven in large trials.

In addition to the changes of microbial gene abundances related to metabolism modules (Figure 3), enrichments of D-alanine metabolism, synthesis/degradation of ketone bodies and glutathione metabolism were noted in CKD-LPD patients. D-alanine metabolism intervenes in the glucose-alanine cycle of gluconeogenesis and participates actively in process of protein synthesis. The ketone bodies are substrates contributing to lipogenesis and sterol biosynthesis in anabolic condition and can also reduce oxidative stress by inhibiting reactive oxygen species production and increasing antioxidant proteins to prevent lipid peroxidation and protein oxidation during periods of starvation [38]. Likewise, gut microbiota interacts, through the modification of substrate availability secondary to protein restriction, with the diet leading to metabolic pathway reprogramming and impacting on host functioning to adapt this nutrient manipulation. Together with the synergistic changes of metabolomic profile observed in patients receiving LPD, the findings of this study illustrated remodeling and adaption of gut microbiota and their genetic potential to compensate possible energy wasting in face of dietary protein restriction in renal patients.

The causality of the association should be interpreted with caution because of the cross-sectional design, small number of patients and limited dietary intervention of only three-months in this study. Several shortcomings should be also addressed, including unique ethnic group, inference of functional capacities of bacterial communities based on 16S rRNA gene sequencing and unavailability of fecal concentration of metabolites to reflect their intestinal generation. However, detailed dietary recall, accurate recording of daily protein intake from 24h urine nitrogen estimates and matching of common confounding characteristics from baseline may all minimize bias of the study and strengthen the conjecture of our supposition. Further prospective longitudinal or randomized studies with breakthrough methodologies, such as shotgun metagenomic sequencing, may help to elucidate the function of LPD intervention on mysterious intestinal microbiome–host metabolite synergies in order to preserve renal function of CKD patients.

## 5. Conclusions

In conclusion, our findings establish a comprehensive understanding in the relationship between the intestinal microbiota and host metabolism in patients receiving dietary protein restriction, a common dietary counseling instructed to CKD patients, providing potential avenues for microbiome-based dietary manipulation and modalities for modulating intestinal dysbiosis to impact on the outcome of renal patients.

## Figures and Tables

**Table 1:** Baseline characteristics of study population (*n* = 77).

	All Patients	Non-CKD	CKD-NPD	CKD-LPD	*p*
	*n* = 77	*n* = 34	*n* = 27	*n* = 16	
Age, mean (SD)	63.40 ± 6.49	62.15 ± 6.58	63.48 ± 6.12	65.94 ± 6.52	0.227
Male, *n* (%)	38 (49.40)	14 (41.20)	13 (48.10)	11 (68.80)	0.267
Diabetes, *n* (%)	41 (53.20)	16 (47.10)	15 (55.60)	10 (62.50)	0.477
Hypertension, *n* (%)	60 (77.90)	21 (61.80)	23 (85.20)	16 (100.00)	0.245
Body mass index	25.82 ± 3.47	25.92 ± 3.82	26.12 ± 3.04	25.10 ± 3.51	0.271
Systolic pressure, mmHg	133.26 ± 17.14	130.88 ± 18.10	135.15 ± 13.50	135.13 ± 20.71	0.891
Dietary intake (serving/day)					
Vegetable	1.7 ± 0.8	1.9 ± 0.8	1.5 ± 0.6	1.0 ± 0.5	0.182
Meat	1.6 ± 0.8	1.8 ± 0.9	1.6 ± 0.8	1.1 ± 0.6	0.274
Fruit	0.7 ± 0.6	0.7 ± 0.5	0.7 ± 0.6	0.6 ± 0.5	0.930
Rice/noodle	2.3 ± 0.7	2.3 ± 0.7	2.5 ± 0.8	2.3 ± 0.9	0.661
Estimated protein intake, g/day	1.00 ± 0.35	1.22 ± 0.41	1.16 ± 0.26	0.65 ± 0.12	<0.001
Blood urea nitrogen, mg/dL	27.82 ± 25.74	14.35 ± 3.81	30.59 ± 23.19	51.75 ± 36.84	0.057
Serum creatinine, mg/dL	1.81 ± 2.00	0.78 ± 0.23	2.08 ± 1.83	3.52 ± 2.94	0.065
Estimated GFR, mL/min/m^2^	69.24 ± 52.15	99.52 ± 56.04	53.22 ± 36.06	31.95 ± 24.31	0.047
Hemoglobin, g/dL	12.71 ± 2.07	13.59 ± 1.22	12.66 ± 1.81	10.94 ± 2.74	0.043
Serum albumin, mg/dL	4.43 ± 0.46	4.54 ± 0.24	4.53 ± 0.37	4.02 ± 0.68	0.012
Serum potassium, mEq/L	4.22 ± 0.47	4.06 ± 0.33	4.31 ± 0.37	4.43 ± 0.71	0.595
Fasting sugar, mg/dL	126.09 ± 45.79	119.88 ± 30.97	132.33 ± 52.62	128.75 ± 59.59	0.788
Total cholesterol, mg/dL	191.75 ± 37.57	197.35 ± 26.09	187.33 ± 46.46	187.31 ± 42.26	0.923
hs-CRP, mg/L ^#^	1.62 (2.02)	1.38 (1.82)	1.35 (3.57)	2.15 (1.55)	0.479
Urine protein-creatinine ratio, g/g ^#^	108.34 (375.72)	77.24 (32.49)	223.95 (819.14)	411.85 (2639.28)	0.471
Urine output, mL/day	2081.3 ± 668.3	2197.3 ± 513.9	2229.6 ± 685.0	1751.3 ± 659.2	0.029

Data are expressed in mean (SD) or median (interquartile range). Estimation of *p* value between LPD vs. NPD by using t-test or median test ^#^; Abbreviation: CKD, chronic kidney disease; GFR, glomerular filtration rate; NPD, normal protein diet; LPD, low protein diet; hs-CRP, high sensitive C reactive protein. Estimated protein intake (g/day) = 6.25 × [Urine urea nitrogen (g/d) + 30 mg/kg/d × Weight(kg)].

**Table 2:** Change of gut microbiota (at genus and species-level) associated with different dietary regimen.

Gut Microbiota		RA (%) Non-CKD	RA (%) CKD-LPD	RA (%) CKD-NPD	*P* *	*p* (LPD vs. NPD) ^#^	*p* (LPD vs. Non-CKD) ^#^	*p* (NPD vs. Non-CKD) ^#^
Family	Genus							
Bacillaceae	Calditerricola ↑	0.0000887	0.00117	0.0000985	0.003579	0.001398	0.001015	0.4818
Desulfovibrionaceae	Desulfovibrio ↓	0.000177	0.0015	0.00405	0.01513	0.4994	0.01308	0.004705
Lachnospiraceae	Pseudobutyrivibrio ↓	4.099	1.878	3.557	0.0007933	0.02095	0.0000888	0.02529
Lachnospiraceae	Lachnospira ↓	0.06535	0.0258	0.07014	0.003283	0.04567	0.0004307	0.03001
Lachnospiraceae	Eubacterium_hallii_group ↓	0.7137	0.3318	0.8227	0.007689	0.01179	0.0009717	0.1778
Lachnospiraceae	Roseburia ↓	0.2302	0.08307	0.1382	0.007848	0.04956	0.001018	0.05064
Lachnospiraceae	Coprococcus_1 ↑	0.00071	0.01715	0.00069	0.01597	0.01435	0.00229	0.2399
Lachnospiraceae	Fusicatenibacter ↓	0.2073	0.1658	0.2177	0.01854	0.006801	0.004211	0.4444
Lachnospiraceae	Anaerostipes ↓	1.653	0.8215	1.58	0.02399	0.01337	0.00408	0.3255
Lachnospiraceae	Lachnoclostridium ↓	0.8864	0.2202	0.8797	0.0005679	0.002618	0.0000611	0.1225
Peptostreptococcaceae	Romboutsia ↑	0.9836	1.486	0.9934	0.04816	0.007215	0.03711	0.2043
Porphyromonadaceae	Parabacteroides ↑	0.000444	0.0015	0.000197	0.02739	0.004277	0.01831	0.2457
Prevotellaceae	Alloprevotella ↑	0.00471	0.03646	0.00543	0.02063	0.005736	0.005909	0.4726
Prevotellaceae	Prevotellaceae_NK3B31 ↓	0.02477	0.000499	0.015	0.02164	0.02963	0.00285	0.1629
Ruminococcaceae	Subdoligranulum ↑	0.0087	0.02014	0.000395	0.0007012	0.000141	0.0757	0.004089
Ruminococcaceae	Ruminococcaceae_UCG-010 ↑	0.000355	0.002	0.00178	0.008634	0.01071	0.001147	0.2054
Ruminococcaceae	Faecalibacterium ↑	0.02628	0.02231	0.0146	0.01278	0.004034	0.2441	0.009593
Ruminococcaceae	Subdoligranulum ↑	0.00648	0.01698	0.00128	0.02464	0.008032	0.2859	0.01378
Synergistaceae	Cloacibacillus ↑	0.00311	0.01282	0.01154	0.02992	0.007619	0.008901	0.452
**Family/genus**	**Species**							
Bacteroidaceae/Bacteroides	Bacteroides_coprophilus ↓	0.4395	0.01698	0.04913	0.003919	0.02987	0.0004587	0.05165
Bacteroidaceae/Bacteroides	Bacteroides_plebeius ↓	2.189	0.3233	0.6128	0.005041	0.1501	0.001213	0.01054
Bacteroidaceae/Bacteroides	Bacteroides_eggerthii ↓	0.22	0.0283	0.2326	0.01589	0.03211	0.001999	0.1235
Clostridiaceae/Clostridium_sensu_stricto_1	Clostridium_paraputrificum ↑	0.01474	0.03363	0.01934	0.003622	0.03818	0.0004482	0.03853
Clostridiaceae/Peptoclostridium	Clostridium_sordellii ↑	0.01385	0.05743	0.03325	0.0001,322	0.03922	0.0000,226	0.003827
Coriobacteriaceae/Olsenella	Olsenella_uli ↑	0.00124	0.00183	0.000394	0.02391	0.004397	0.1401	0.03176
Eubacteriaceae/Mogibacterium	Mogibacterium_diversum ↑	0.000621	0.00216	0.000789	0.01012	0.004809	0.002067	0.3947
Lachnospiraceae/Blautia	Blautia_hydrogenotrophica ↑	0.08488	0.09689	0.0731	0.03329	0.009464	0.008729	0.493
Lactobacillaceae/Lactobacillus	Lactobacillus_mucosae ↑	0.04377	1.765	0.07665	0.01466	0.01764	0.001946	0.1935
Porphyromonadaceae/Porphyromonas	Porphyromonas_gingivalis ↑	0.000355	0.01731	0.000789	0.02485	0.04228	0.00328	0.1312
Streptococcaceae/Streptococcus	Streptococcus_anginosus ↑	0.05727	0.4275	0.07586	0.009104	0.004643	0.001801	0.3808
Veillonellaceae/Megasphaera	Lactobacillus_sp._AB032 ↑	0.01394	0.1806	0.00641	0.03676	0.01011	0.009835	0.4841

Abbreviation: RA, relative abundance; CKD, chronic kidney disease; LPD, low protein diet; NPD, normal protein diet. * *p* value among three groups by using Kruskal–Wallis test; ^#^ *p* value between two groups by using Dunn’s test. ↑ and ↓ indicate an increase or decrease in bacterial abundance associated with LPD compared to NPD.

**Table 3:** Correlation between relative abundances of microbes and daily protein intake.

	Overall Patients		Only CKD Patients	
	r	*p*	r	*p*
**Genus**				
Anaerostipes	0.343	0.009	0.346	0.023
Calditerricola	−0.278	0.036	−0.336	0.027
Eubacterium hallii group	0.263	0.048	0.371	0.014
**Species**				
Streptococcus anginosus	−0.466	<0.001	−0.442	0.003
Lactobacillus mucosae	−0.4	0.002	−0.409	0.006
Clostridium paraputrificum	−309	0.019	−0.446	0.003
r = rho-based Spearman correlation coefficient				

**Table 4:** Characteristics of resampling subset of patients with individualized match to renal function and age.

	rCKD-NPD	rCKD-LPD	*p*
Age, mean (SD)	65.14 ± 6.5	64.86 ± 5.3	0.890
Male, *n* (%)	5 (35.7%)	9 (64.3%)	0.131
Diabetes, *n* (%)	8 (57.1%)	9 (64.3%)	0.699
Estimated GFR, mL/min/m^2^	40.58 ± 23.3	30.22 ± 22.8	0.240
Serum creatinine, mg/dL	2.42 ± 2.1	3.66 ± 3.1	0.220
Estimated protein intake, g/day	1.22 ± 0.3	0.67 ± 0.1	<0.001
Urine protein-creatinine ratio, g/g ^#^	465.85 (1952.44)	264.41 (2241.62)	0.950
Urine output, mL/day	2182.14 ± 721.5	1739.29 ± 697.6	0.110
**Genus, relative abundance (%)**			
Anaerostipes	1.77 ± 0.0,142	0.9 ± 0.006	0.047
Calditerricola	0.0001 ± 0.0,001	0.001 ± 0.002	0.050
Eubacterium hallii group ^#^	3.04 ± 0.0,238	1.81 ± 0.0105	0.050
**Species, relative abundance (%)**			
Streptococcus anginosus ^#^	0.06 ± 0.0,007	0.47 ± 0.0121	0.041
Lactobacillus mucosae ^#^	0.1 ± 0.0,012	2.02 ± 0.0498	0.035
Clostridium paraputrificum ^#^	0.01 ± 0.0,001	0.04 ± 0.0004	0.060
**Metabolites**			
Glyco-λ-muricholic acid, nmol/L ^#^	9.75 (5.04)	13.93 (18.5)	0.011
Acetic acid, mg/L ^#^	1.69 (0.94)	1.58 (0.31)	0.860
Heptanoic acid, mg/L ^#^	0.33 (1.04)	0.09 (0.36)	0.176
Nonanoic acid, mg/L ^#^	0.12 (2.76)	0.08 (0.03)	0.005
Total indoxyl sulfate, mg/L ^#^	2.7 (5.87)	4.54 (13.84)	0.257
Total p-cresyl sulfate, mg/L ^#^	8.66 (23.76)	5.45 (20.5)	0.946
Free indoxyl sulfate, mg/L ^#^	0.26 (0.94)	0.34 (1.56)	0.257
Free p-cresyl sulfate, mg/L ^#^	0.64 (1.23)	0.39 (1.30)	0.796

Data are expressed in mean (SD) or median (interquartile range). Estimation of *p* value between LPD vs. NPD by using chi-square, t-test or median test ^#^. Abbreviation: rCKD, resampling subset of chronic kidney disease; NPD, normal protein diet; LPD, low protein diet.

## Supplementary Materials

The following are available online at https://www.mdpi.com/2072-6643/12/9/2799/s1, Table S1: Changes of serum SCFA and MCFA concentration associated with different dietary regimens (Mean ± SD); Table S2: Changes of serum bile acids concentration associated with different dietary regimens (Mean ± SD); Figure S1: Study design and patient flow schema; Figure S2: Determinations of best discriminatory bacterial taxa associated with CKD-LPD. Best discriminatory taxa categorizing CKD-LPD were estimated by performing Random Forests analysis using the overall OTU (**A**) only genus-levels abundances (**B**) or only species-level abundances (**C**) datasets against the dietary regimens. Bacterial taxa that are most discriminatory were ranked in decremental order of their importances to the accuracy of the model. Importance was evaluated based on the mean decrease in accuracy of microbiota prediction when the relative abundance of each taxon was randomly permuted. LPD, low protein diet; NPD, normal-protein diet.
