# Supplementary material for: Compositional and Functional Adaptations of Intestinal Microbiota and Related Metabolites in CKD Patients Receiving Dietary Protein Restriction
Source: Nutrients. 2020 Sep 12;12(9):2799. doi: 10.3390/nu12092799 (PMC7551076; doi:10.3390/nu12092799)
Supplement: Supplementary file 1 [file nutrients-12-02799-s001.pdf]

Table S1. Changes of serum SCFA and MCFA concentration associated with different dietary regimens (Mean ± SD).

Fatty acids (mg/L)	Molecular formula	Non-CKD	CKD-LPD	CKD-NPD	P*	P(LPD vs. NPD)#	P (LPD vs. non-CKD)#	p (NPD vs. non-CKD)#
Acetic acid	$C_2H_4O_2$	2.03 ± 0.37	1.91 ± 1.07	1.76 ± 0.53	0.018	0.818	0.1	0.236
Propionic acid	$C_3H_6O_2$	1.19 ± 0.26	1.01 ± 0.43	1.03 ± 0.37	0.165	0.818	0.347	0.554
Isobutyruc acid	$C_4H_8O_2$	0.31 ± 0.07	0.51 ± 0.83	0.65 ± 0.77	0.673	0.34	0.347	1
Butyric acid	$C_4H_8O_2$	1.68 ± 0.46	1.57 ± 0.64	1.51 ± 0.78	0.953	0.818	0.812	0.554
Isovaleric acid	$C_5H_{10}O_2$	0.04 ± 0.01	0.05 ± 0.02	0.06 ± 0.03	0.341	0.34	0.347	1
Valeric acid	$C_5H_{10}O_2$	0.13 ± 0.03	0.12 ± 0.04	0.12 ± 0.03	0.563	0.818	0.812	1
Hexanoic acid	$C_6H_{12}O_2$	0.11 ± 0.03	0.12 ± 0.11	0.16 ± 0.11	0.154	0.093	0.1	1
Heptanoic acid	$C_7H_{14}O_2$	0.07 ± 0.09	0.42 ± 0.75	0.41 ± 0.54	0.004	0.818	0.243	0.018
Octanoic acid	$C_8H_{16}O_2$	0.14 ± 0.05	0.12 ± 0.08	0.19 ± 0.14	0.248	0.34	0.347	0.554
Nonanoic acid	$C_9H_{18}O_2$	0.1 ± 0.02	0.38 ± 1.05	0.75 ± 1.39	0.002	0.002	0.1	0.018
Decanoic acid	$C_{10}H_{20}O_2$	0.34 ± 0.12	0.25 ± 0.13	0.38 ± 0.24	0.085	0.818	0.347	1

Abbreviation: SCFA, short-chain fatty acids; MCFA, medium-chai fatty acids; CKD, chronic kidney disease; LPD, low protein diet; NPD, normal protein diet. * p value among three groups by using Kruskal–Wallis test; # p value between two groups by using Median test.

Table S2. Changes of serum bile acids concentration associated with different dietary regimens (Mean ± SD).

**Bile acids (mM)**	**Molecular formula**	**Non-CKD**	**CKD-LPD**	**CKD-NPD**	**P***	**p (LPD vs. NPD)#**	**p (LPD vs. non-CKD)#**	**p (NPD vs. non-CKD)#**
12-Dehydrocholic acid	C24H38O5	10.93 ± 5.8	10.59 ± 2.82	11.73 ± 4.14	0.272	0.748	0.817	0.247
12-Ketolithocholic acid	C24H38O4	11.88 ± 12.33	13.41 ± 17.16	21.95 ± 34.89	0.94	0.488	0.347	1
23-Nordeoxycholic acid	C23H38O4	2.31 ± 2.18	2.68 ± 2.45	2.94 ± 2.54	0.548	0.974	0.487	0.65
3-Dehydrocholic acid	C24H38O5	3.69 ± 4.42	3.07 ± 1.55	3.56 ± 2.28	0.458	0.73	0.901	0.299
7-Ketodeoxycholic acid	C24H38O5	7.47 ± 7.66	33.53 ± 94.7	6.71 ± 2.81	0.479	0.701	0.44	0.305
7-Ketolithocholic acid	C24H38O4	20.62 ± 22.74	24.4 ± 16.41	31.05 ± 35.11	0.473	0.866	0.648	0.959
Allocholic acid	C24H40O5	5.49 ± 1.69	6.7 ± 3.74	6.67 ± 2.69	0.268	0.914	0.598	0.165
Chenodeoxycholic acid	C24H40O4	642.97 ± 870.73	493.74 ± 701.46	691.12 ± 793.99	0.677	0.818	0.812	1
Cholic acid	C24H40O5	229.98 ± 409.87	407.96 ± 879.26	203.93 ± 201.53	0.388	0.34	0.642	0.076
Deoxycholic acid	C24H40O4	458.08 ± 459.29	663.06 ± 1045.48	534.8 ± 600.18	0.927	0.715	0.724	0.762
Glycochenodeoxycholic acid	C26H43NO5	2536.55 ± 5981.16	885.28 ± 930.32	1339.52 ± 2181.26	0.623	0.818	0.812	0.554
Glycocholic acid	C26H43NO6	1230.9 ± 4335.65	211.35 ± 245.11	832.64 ± 3237.66	0.591	0.622	0.347	0.236
Glycodeoxycholic acid	C26H43NO5	632.8 ± 1558.89	304.44 ± 246.43	1208.18 ± 4030.28	0.913	0.818	0.642	1
Glycohyodeoxycholic acid	C26H43NO5	1.78 ± 1.29	2.11 ± 1.85	3.55 ± 6.13	0.969	0.73	0.732	0.74
Glycolithocholic acid	C26H43NO4	9.05 ± 10.33	4.25 ± 3.73	15.72 ± 19.32	0.157	0.363	0.156	0.825
Glycoursodeoxycholic acid	C26H43NO5	329.25 ± 844.67	197.84 ± 259.28	235.1 ± 255.23	0.557	0.818	0.812	0.236
Glyco-λ-muricholic acid	C26H43NO6	28.85 ± 42.72	19.43 ± 16.99	17.95 ± 39.82	0.081	0.027	0.812	0.067
Hyodeoxycholic acid	C24H40O4	5.24 ± 5.73	4.81 ± 3.63	8.33 ± 13.85	0.99	0.918	0.794	0.873

Isolithocholic acid	C24H40O3	14.02 ± 8.42	11.55 ± 6.16	16.24 ± 11.24	0.298	0.234	0.35	0.944
Lithocholic acid	C24H40O3	15.47 ± 11.16	12.7 ± 7.22	16.74 ± 9.15	0.286	0.161	0.557	0.437
Taurochenodeoxycholic acid	C26H45NO6S	585 ± 1353.24	162.58 ± 142.27	194.16 ± 190.72	0.697	0.818	0.812	1
Taurocholic acid	C26H45NO7S	574.86 ± 2184.64	44.55 ± 58	246.28 ± 881.83	0.895	0.818	0.724	0.889
Taurodeoxycholic acid	C26H45NO6S	90.27 ± 205.45	44.63 ± 41.13	110.65 ± 289.71	0.75	0.34	0.642	0.236
Taurolithocholic acid	C26H45NO5S	9.77 ± 0	9.77 ± 0	10.24 ± 2.13	0.414	0.773	NA	0.93
Tauroursodeoxycholic acid	C26H45NO6S	23.52 ± 33.23	17.37 ± 14.53	19.17 ± 17.58	0.847	0.914	0.732	0.71
Ursocholic acid	C24H40O5	7.39 ± 8.94	7.52 ± 6.37	8.15 ± 6.99	0.359	0.73	0.367	0.282
Ursodeoxycholic acid	C24H40O4	126.87 ± 153.22	149.54 ± 183.57	238.93 ± 374.06	0.484	0.818	0.812	0.554
β-Muricholic acid	C24H40O5	2.55 ± 0.49	2.45 ± 0	2.8 ± 0.88	0.229	0.457	0.704	0.479
λ-Muricholic acid	C24H40O5	25.48 ± 12.3	31.39 ± 17.55	30.34 ± 14.99	0.39	0.866	0.474	0.476
α-Muricholic acid	C24H40O5	UD	UD	UD	–	–	–	–
6,7-Diketolithocholic acid	C24H36O5	UD	UD	UD	–	–	–	–
7,12-Diketolithocholic acid	C24H36O5	UD	UD	UD	–	–	–	–
Allolithocholic acid	C24H40O3	UD	UD	UD	–	–	–	–
Apocholic acid	C24H38O4	UD	UD	UD	–	–	–	–
Dehydrocholic acid	C24H34O5	UD	UD	UD	–	–	–	–
Dehydrolithocholic acid	C24H38O3	UD	UD	UD	–	–	–	–
Isodeoxycholic acid	C24H40O4	UD	UD	UD	–	–	–	–
Tauro α-Muricholic acid	C26H45NO7S	UD	UD	UD	–	–	–	–
Tauro β-Muricholic acid	C26H45NO7S	UD	UD	UD	–	–	–	–
Taurohyodeoxycholic acid	C26H45NO6S	UD	UD	UD	–	–	–	–

Glycodehydrocholic acid	$C_{26}H_{37}NO_6$	UD	UD	UD	–	–	–	–

Abbreviation: CKD, chronic kidney disease; LPD, low protein diet; NPD, normal protein diet; UD, undetectable. * p value among three groups by using Kruskal–Wallis test; # p value between two groups by using Median test.

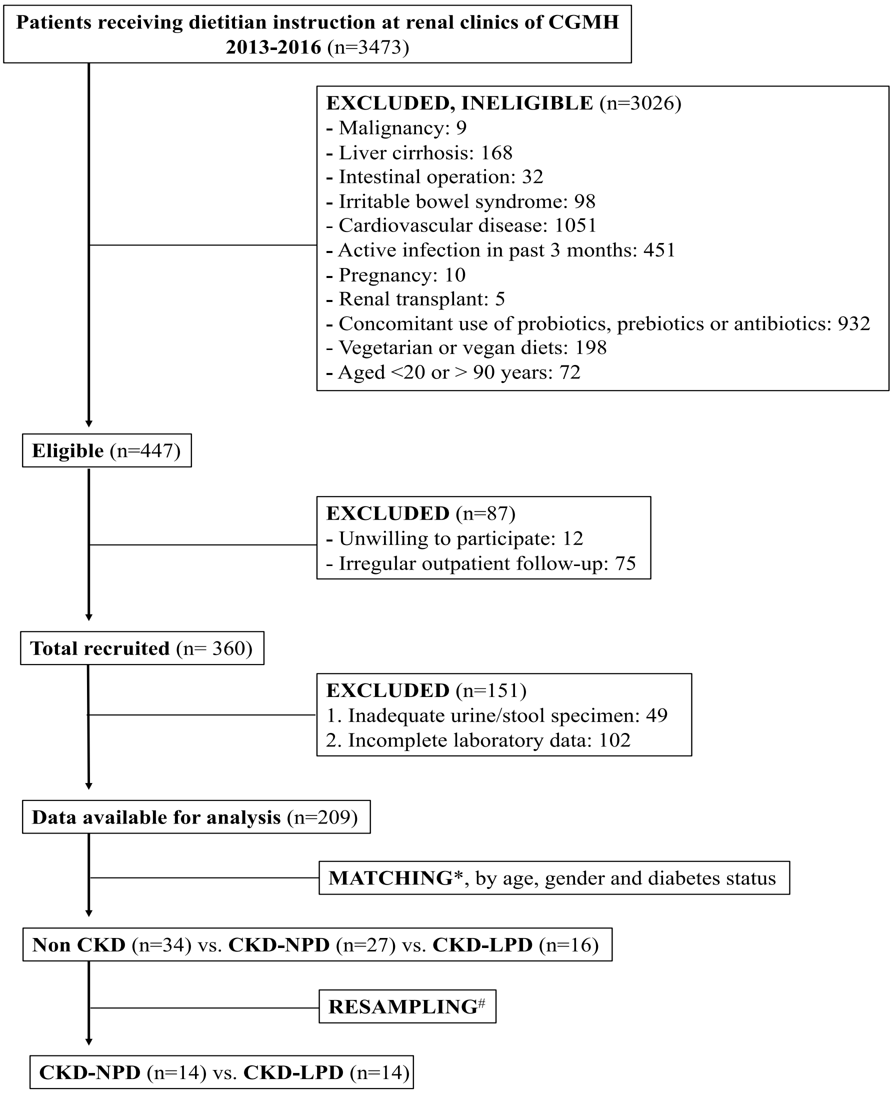

Abbreviation: CGMH, Chang Gung Memorial Hospital at Keelung; CKD, chronic kidney disease; NPD, normal-protein diet; LPD, low-protein diet.

Note: *Matching by age, gender and comorbidities (diabetes and hypertension); #Resampling of patients by individualized matching in terms age ± 1 year, gender and CKD stage.

Figure 1. Study design and patient flow schema.

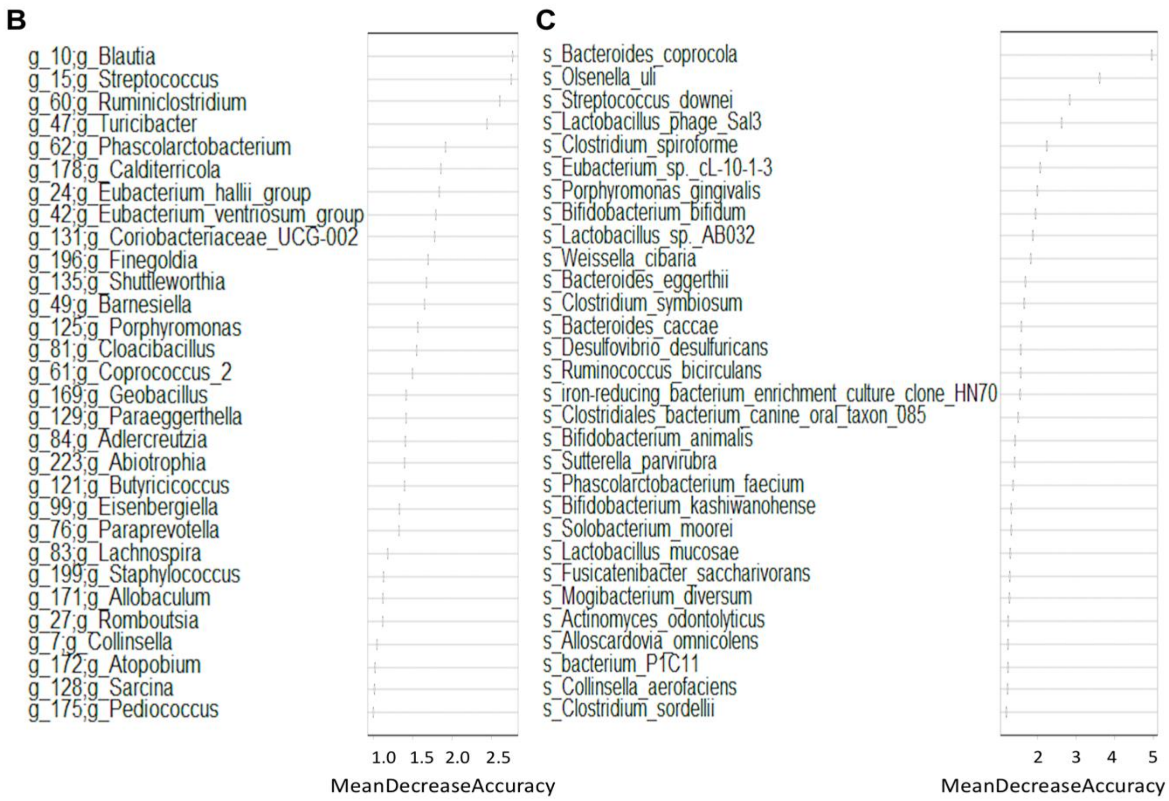

Figure S2. Determinations of best discriminatory bacterial taxa associated with CKD-LPD. Best discriminatory taxa categorizing CKD-LPD were determined by applying Random Forests analysis using the overall OTU **(A)**, only genus-levels abundances **(B)** or only species-level abundances **(C)** datasets against the dietary regimens**.** Bacterial taxa that are most discriminatory were ranked in descending order of their importances to the accuracy of the model. Importance was determined based on the mean decrease in accuracy of microbiota prediction when the relative abundance of each taxon was randomly permuted. LPD, low protein diet; NPD, normal-protein diet.
